# Disseminated BCGosis After Intravesical BCG Installation for Bladder Cancer

**DOI:** 10.1155/crdi/9532859

**Published:** 2025-09-26

**Authors:** Tyler Martinson, C. Bradley Hare, Andrea Kirmaier, Joseph C. Presti, John L. Shaia, Bian Wu, Jonathan E. Volk

**Affiliations:** ^1^Department of Internal Medicine, Kaiser Permanente, San Francisco, California, USA; ^2^Department of Infectious Diseases, Kaiser Permanente, San Francisco, California, USA; ^3^Department of Pathology, The Permanente Medical Group, Berkeley, California, USA; ^4^Department of Urology, Kaiser Permanente, San Francisco, California, USA; ^5^Department of Oncology, Kaiser Permanente, San Francisco, California, USA; ^6^Department of Vascular Surgery, Kaiser Permanente, San Francisco, California, USA

**Keywords:** Bacillus Calmette–Guérin, BCG immunotherapy complications, bladder cancer, disseminated BCGosis, mycobacterial infection

## Abstract

We present three patients who developed BCGosis, a rare complication following intravesical Bacillus Calmette–Guérin (BCG) treatment for bladder cancer. These cases highlight the diverse clinical manifestations, diagnostic challenges, and management strategies of this entity, emphasizing the importance of early recognition and initiation of antimycobacterial treatment.

## 1. Introduction

Intravesical Bacillus Calmette–Guérin (BCG) immunotherapy is considered a cornerstone in the management of nonmuscle invasive bladder cancer (NMIBC). First described in 1976 [1], this immunotherapeutic approach utilizes a live, attenuated strain of *Mycobacterium bovis*, a part of the Mycobacterium tuberculosis complex (MTBC), to stimulate immune reactivity which inhibits tumor growth and reduces risk of tumor progression and recurrence following transurethral resection of bladder tumors (TURBT). Numerous meta-analyses have affirmed the superior efficacy of adjunctive intravesical BCG installation compared to TURBT alone or TURBT in conjunction with chemotherapy [2–4], positioning BCG immunotherapy as part of the standard of care in NMIBC management [5–7]. However, this attenuated *M. bovis* treatment can result in severe, disseminated BCG infection, a clinical entity known as BCGosis.

**Figure 1:**
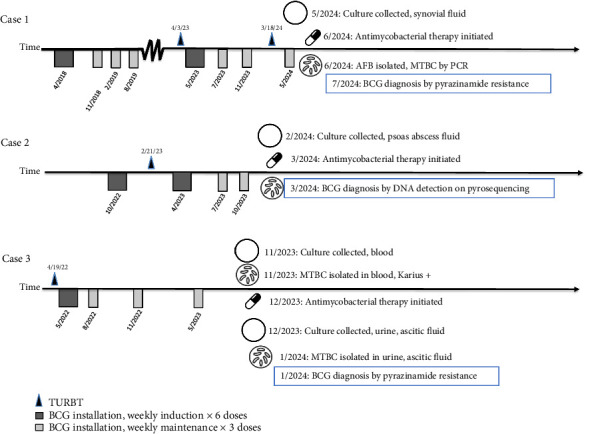
Pictorial representation of bladder cancer treatment and BCG diagnostic and therapeutic course.

BCGosis occurs in 3%–7% of patients undergoing BCG therapy and may be localized in the genitourinary system (23.4%) or present with musculoskeletal (19.9%), vascular (6.7%), or disseminated (34.4%) symptoms [8–10]. Understanding the spectrum of BCGosis is crucial, as the diversity of clinical presentations poses challenges in diagnosis and management. In this case series, we present three patients who received adjunctive BCG immunotherapy for bladder cancer and subsequently developed different manifestations of BCGosis between December 2023 and May 2024 at a single medical center in San Francisco, California (Figure 1).

## 2. Case Presentations

### 2.1. Case 1

A 75-year-old male with seronegative rheumatoid arthritis, on methotrexate, and bilateral knee osteoarthritis was diagnosed with carcinoma in situ (CIS) of the bladder. Beginning in April 2018, the patient received induction therapy with BCG, administered weekly for six weeks followed by maintenance BCG therapy with three weekly installations occurring at Months 3, 6, and 12 postinduction. Recurrent CIS was confirmed by TURBT in April 2023, and retreatment with BCG was initiated in May 2023, following the same induction and maintenance schedule previously used. In June 2023, the patient had a negative QuantiFERON-TB Gold Plus (QFT-Plus) test result [11]. In March 2024, he underwent a second TURBT, which revealed negative biopsies for malignancy.

The patient presented in May 2024 in consultation for elective total knee arthroplasty after three weeks of worsening left knee pain, edema, and reduced mobility. A repeat QFT-Plus test produced indeterminate results due to a lack of response to both mitogen control and TB-specific antigens, attributed to his immunosuppressive therapy. Inflammatory markers (ESR/CRP) were elevated, prompting a knee joint aspiration, which demonstrated acid-fast bacilli (AFB) in synovial fluid culture, later identified as MTBC by polymerase chain reaction (PCR). Prior to species identification of the MTBC, the patient was started on rifampin, isoniazid (with pyridoxine), pyrazinamide, and ethambutol, with levofloxacin also added given concerns for BCGosis.

In July 2024, he developed significant hepatotoxicity, presenting with encephalopathy and cholestatic liver enzyme abnormalities, attributed to his medications. Soon after, pyrazinamide resistance was reported from the synovial fluid culture analysis, and whole genome sequencing (WGS) confirmed the presence of *M*. *bovis* BCG in the left knee joint aspirate. In August 2024, due to recurrent drug toxicities, the patient was transitioned to ethambutol, levofloxacin, and linezolid for a planned 12-month course of targeted therapy.

### 2.2. Case 2

An 85-year-old male was diagnosed with NMIBC of the bladder neck. Following the completion of 6-week induction therapy with intravesical BCG, a TURBT was performed in February 2023, revealing multifocal high-grade Ta tumors that were resected. One month later, the patient began a second BCG induction protocol consisting of six weekly intravesical installations, followed by three weekly maintenance regimens at Months 3 and 6.

**Figure 2:**
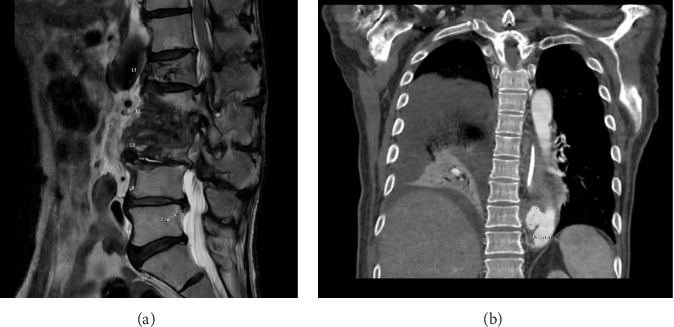
(a) (Case 2) Imaging showing L2-L3 osteomyelitis–discitis, with epidural phlegmons and paraspinal abscesses; (b) (Case 3) imaging showing large right basilar lung consolidation and mycotic aneurysm of the descending thoracic aorta.

Four months after his second maintenance BCG installation, the patient began to experience worsening lower back pain, which was initially attributed to his known lumbar facet arthropathy. A fall related to this pain resulted in hospitalization where he was found to have L2-L3 osteomyelitis–discitis, accompanied by epidural phlegmons, paraspinal abscesses, and bilateral psoas abscesses (Figure 2).

Initial bacterial cultures of blood and psoas fluid aspirate collected in February 2024 were negative. The patient was treated with broad-spectrum antibiotics and discharged to a skilled nursing facility to complete a 6-week, empiric antibiotic course. Subsequently, in March 2024, his psoas abscess culture grew MTBC. He was readmitted, ruled out for pulmonary tuberculosis, and started on rifampin, isoniazid (with pyridoxine), pyrazinamide, and ethambutol. CT imaging revealed persistent, large fluid collections in the bilateral psoas abscesses, necessitating the placement of bilateral drains.

Due to significant transaminitis, all antimicrobials were held within the first week of hospitalization. Pyrosequencing later confirmed a diagnosis of disseminated BCG infection by detection of *M. bovis* in the psoas abscess aspirate, and the treatment resumed on hospital Day 14 with rifabutin, ethambutol, and levofloxacin therapy, with plans for at least a 12-month course of therapy.

Despite these interventions, the patient's condition deteriorated, with worsening anemia, liver function test derangements, and reduced mobility due to increasing pain. His infection progressed despite treatment, and he died after transitioning to comfort care.

### 2.3. Case 3

A 72-year-old male with history of colon cancer and high-grade T1 bladder cancer involving both ureteral orifices underwent partial bilateral resection via TURBT in April 2022. Following this, he began 6-week induction therapy with intravesical BCG in May 2022, with maintenance treatments at Months 3, 6, and 12.

After his Month 6 maintenance cycle of intravesical BCG, he was found to have metasynchronous small bowel adenocarcinoma, and neoadjuvant nivolumab/ipilimumab were started. He developed a Grade 3 skin rash and pneumonitis, necessitating a 3-month prednisone taper, and underwent a Whipple procedure. During the following month, and seven months following his last maintenance BCG treatment, he was admitted to the hospital with a fever and ascites and was found to have transaminitis, pancytopenia, a large right basilar lung consolidation, and multiple enlarging mycotic thoracic and thoracoabdominal aortic pseudoaneurysms (Figure 2). Molecular testing confirmed the presence of MTBC in blood, urine, and ascitic fluid, and the urine isolate was later confirmed as*M. bovis* BCG by PCR andby phenotypic pyrazinamide resistance.

The patient started levofloxacin, amikacin, rifampin, and linezolid to treat his disseminated BCGosis. He was later transitioned to isoniazid (with pyridoxine), rifabutin, and ethambutol, with the plan to complete at least an 18-month course to manage the disseminated infection that involved the bone marrow, liver, peritoneum, lung, vascular structures, and bladder.

After treatment initiation, the patient's symptoms improved significantly, with reduction in fever and improvement of leukopenia, transaminitis, and ascites. The patient ultimately underwent multiple endovascular aortic repairs (TEVAR and fenestrated EVAR) with rifampin-soaked endografts for his mycotic aneurysms. Patient 3 continues treatment, with a plan for life-long antibiotic suppression to protect his aortic endografts.

## 3. Discussion

We present a case series of three patients who developed disseminated BCGosis following intravesical BCG immunotherapy for NMIBC, highlighting the various clinical presentations, diagnostic challenges, and management strategies associated with this rare but serious adverse effect of BCG therapy. These patients lacked risk factors for *M. tuberculosis* and received BCG installations from different batches. When MTBC is identified in patients who have received BCG treatment, especially in those without tuberculosis risk factors, additional diagnostic testing for BCGosis is critical.

BCGosis can manifest in a variety of ways, including localized genitourinary disease, as well as systemic involvement affecting the musculoskeletal, hepatic, pulmonary, vascular, and hematologic systems [8–10]. Here, we observed symptoms ranging from focal septic arthritis to severe multisystem involvement. This variability in clinical manifestations necessitates a high index of suspicion for BCGosis in patients treated with intravesical BCG who present with either systemic or localized symptoms, regardless of the timing relative to their BCG treatment.

The diagnostic process for disseminated BCGosis can be complex. It often requires microbiological cultures, molecular analyses, imaging studies, and histopathological confirmation, which may not be immediately available. In our series, the diagnoses were confirmed through cultures of blood, urine, abscess fluid, ascitic fluid, and synovial fluid, which required two to six weeks for MTBC identification, which prompted further molecular analyses and phenotypic susceptibility testing. Molecular*M. bovis* BCG confirmation was performed via WGS or targeted next-generation sequencing (tNGS), analyzing 16S rRNA, single-nucleotide polymorphisms (SNPs), orspecific DNA regions (e.g., RD1 is absent in BCG strains but present in other MTBC). Pyrazinamide resistance can indirectly confirm *M. bovis/M. bovis* BCG, as it is an intrinsic property of both. If co-infection with *M. tuberculosis* and BCG is suspected, QFT-Plus testing may be useful, as it does not react to *M. bovis* BCG [11].

Optimal antimycobacterial agents and duration for treatment of BCGosis entails a 2-month initiation phase of isoniazid, rifampin, and ethambutol, followed by an extended continuation phase of isoniazid and rifampin, once drug susceptibilities have been confirmed, for a total of at least nine months of treatment [12, 13]. Antimycobacterial selection was guided by known BCG resistance to pyrazinamide and expert consensus on disseminated BCG infection. Drug changes (e.g., to rifabutin and levofloxacin) were based on toxicity, clinical progression, and pharmacokinetic profiles. Longer treatment durations (> 12 months) were used due to bone, vascular, or multiorgan involvement, per limited case-based guidance [9].

Risk factors for disseminated BCGosis remain incompletely defined. Immunosuppression and older age may be significant risk factors for complications [9]. Genetic susceptibility may also play a role, though host genetic studies are lacking. All patients in this series were elderly males, two received immunosuppressive therapy, and there was no report of traumatic catheterizations or unforeseen procedural complications among the cases. Despite the absence of preventive measures to reduce infection, it is imperative that clinicians appropriately stratify patients' candidacy and timing of BCG therapy based on individual risk profiles, with caution advised for patients with potential for urogenital epithelium disruption and vigilant monitoring for early signs of complications in patients receiving BCG. While BCGosis can be attributed to disruptions in the uroepithelium, the risk of developing severe complications does not appear to correlate with the specific BCG strain, dosage, number of instillations, or the interval since the initial surgery [9].

In conclusion, disseminated BCGosis is a rare but potentially life-threatening complication of intravesical BCG installation used as adjunctive therapy for bladder cancer. Our cases align with the existing literature describing delayed diagnosis, varied clinical presentations, and high morbidity in disseminated cases [9]. Early recognition, prompt diagnosis, and tailored medication management are crucial for improving patient outcomes. A multidisciplinary approach involving urology, infectious disease, and multiple other subspecialties is often needed. Further research is essential to clarify the underlying mechanisms for disease progression, identify additional risk factors, and establish optimal treatment protocols for this uncommon condition.

## References

[1] Morales A., Eidinger D., Bruce A. W. (1976). Intracavitary Bacillus Calmette-Guerin in the Treatment of Superficial Bladder Tumors. *The Journal of Urology*.

[2] Han R. F., Pan J. G. (2006). Can Intravesical Bacillus Calmette-Guérin Reduce Recurrence in Patients With Superficial Bladder Cancer? A Meta-Analysis of Randomized Trials. *Urology*.

[3] Shelley M. D., Kynaston H., Court J. (2001). A Systematic Review of Intravesical Bacillus Calmette‐Guérin plus Transurethral Resection vs Transurethral Resection Alone in Ta and T1 Bladder Cancer. *BJU International*.

[4] Shelley M. D., Wilt T. J., Court J., Coles B., Kynaston H. (2004). Intravesical bacillus Calmette‐Guérin is Superior to Mitomycin C in Reducing Tumour Recurrence in High‐Risk Superficial Bladder Cancer: A Meta‐Analysis of Randomized Trials. *BJU International*.

[5] Flaig T. W., Spiess P. E., Agarwal N. (2020). Bladder Cancer, Version 3.2020, NCCN Clinical Practice Guidelines in Oncology. *Journal of the National Comprehensive Cancer Network*.

[6] Babjuk M., Burger M., Capoun O. (2022). European Association of Urology Guidelines on Non-Muscle-Invasive Bladder Cancer (Ta, T1, and Carcinoma in Situ). *European Urology*.

[7] Holzbeierlein J., Bixler B. R., Buckley D. I. (2024). Diagnosis and Treatment of Non-Muscle Invasive Bladder Cancer: AUA/SUO Guideline: 2024 Amendment. *The Journal of Urology*.

[8] Lamm D. L., Van Der Meijden A. P., Morales A. (1992). Incidence and Treatment of Complications of Bacillus Calmette-Guerin Intravesical Therapy in Superficial Bladder Cancer. *The Journal of Urology*.

[9] Pérez-Jacoiste Asín M. A., Fernández-Ruiz M., López-Medrano F. (2014). Bacillus Calmette-Guérin (BCG) Infection Following Intravesical BCG Administration as Adjunctive Therapy for Bladder Cancer: Incidence, Risk Factors, and Outcome in a Single-Institution Series and Review of the Literature. *Medicine*.

[10] Arsuffi S., Cambianica A., Di Filippo E. (2023). Vascular Graft Infections Caused by Mycobacterium bovis BCG After BCG Immunotherapy for Non-Muscle-Invasive Bladder Cancer: Case Report and Review of Literature. *Journal of Clinical Tuberculosis and Other Mycobacterial Diseases*.

[11] (2023). QIAGEN QuantiFERON-TB Gold plus (QFT-Plus) Package Insert. 11. QIAGEN.

[12] Zha B. S., Nahid P. (2019). Treatment of Drug-Susceptible Tuberculosis. *Clinics in Chest Medicine*.

[13] Nahid P., Dorman S. E., Alipanah N. (2016). Official American Thoracic Society/Centers for Disease Control and Prevention/Infectious Diseases Society of America Clinical Practice Guidelines: Treatment of Drug-Susceptible Tuberculosis. *Clinical Infectious Diseases*.

